# Inappropriate Defibrillator Shocks due to Mechanical Inference from an Investigational Device

**DOI:** 10.1155/2019/2810396

**Published:** 2019-01-06

**Authors:** Ying chi Yang, Thein Tun Aung, Abdul Wase

**Affiliations:** ^1^University of Iowa Hospitals and Clinics, Iowa City, IA, USA; ^2^Dayton Heart and Vascular Hospital, Dayton, OH, USA

## Abstract

Cardiac contractility modulation (CCM) is an investigational device-based therapy to enhance ventricular contractility in systolic heart failure patients who are not candidates for cardiac resynchronization therapy (CRT) owing to the absence of wide QRS complexes or who have failed to respond on CRT. The principal mechanism is based on the stimulation of cardiac muscles by nonexcitatory electrical signals to augment the influx of calcium ions into the cardiomyocytes. The majority of patients receiving CCM therapy have concurrent implantable cardioverter defibrillators, and the manufacturer declares both devices can be used in parallel without any interactions. Nevertheless, proper lead positioning of both devices are crucial, and it is mandatory to check device-device interactions during each and every cardiac electronic implantable device-related procedure to prevent adverse outcomes.

## 1. Introduction

Cardiac contractility modulation (CCM) can be attained by an investigational device designed to improve the strength of myocardial contractility by delivering nonexcitatory electrical signals to increase calcium ion influx into the cardiomyocytes during the absolute refractory period of the cardiac cycle. The CCM device, Optimizer, is accepted in the United States as an investigational device to treat systolic heart failure patients with narrow QRS complexes or who are nonresponders of cardiac resynchronization therapy (CRT). A CCM unit includes a pulse generator and three pace-sense leads; a sensing lead in the right atrium and two signal-delivering leads implanted at the right ventricular (RV) septum. The manufacturer declares that CCM devices work in parallel with any kinds of implantable cardioverter defibrillator (ICD) system without affecting the ICD function. We present a case in which CCM leads chatter with the ICD lead in the RV which resulted in inappropriate sensing of the CCM signals by the ICD with subsequent delivery of inappropriate shocks.

## 2. Case Presentation

A 72-year-old male with past medical history of myocardial infarction status postpercutaneous coronary intervention, chronic systolic heart failure secondary to ischemic cardiomyopathy, received a single chamber Biotronik implantable cardioverter defibrillator (ICD) with a Medtronic single-coil defibrillator lead (model 6949-65) in 2005. A year later, the patient also received an investigational device, Optimizer III (Impulse Dynamics, Orangeburg, NY) (model #CCMX8), assembled with St. Jude Medical active fixation pace-sense leads (Model 1388-T), one lead in the right atrium and two in the upper and lower interventricular septum. Prominent electrical signals were identifiable on the patient's surface EKG when CCM was activated.

In August 2014, the old Medtronic lead (Model 6949-65) was found to have a sudden increase in RV impedance to >2000 Ohms with “RED” alert warning of the Biotronik device. The particular Medtronic lead was also a subject of a Class-I recall by the Food and Drug Administration. Subsequently, he underwent laser lead extraction and replacement for a new ICD lead, Medtronic Sprint Quattro (Model 6947) dual coil defibrillation lead. The procedure was uneventful. The function of the new ICD system was assessed, and all the device parameters were found within normal limits. Patient's CCM was eight years old at that time and was found no longer functional due to battery depletion. Hence, the device-device interaction with the concurrent CCM unit was not evaluated.

**Figure 1:**
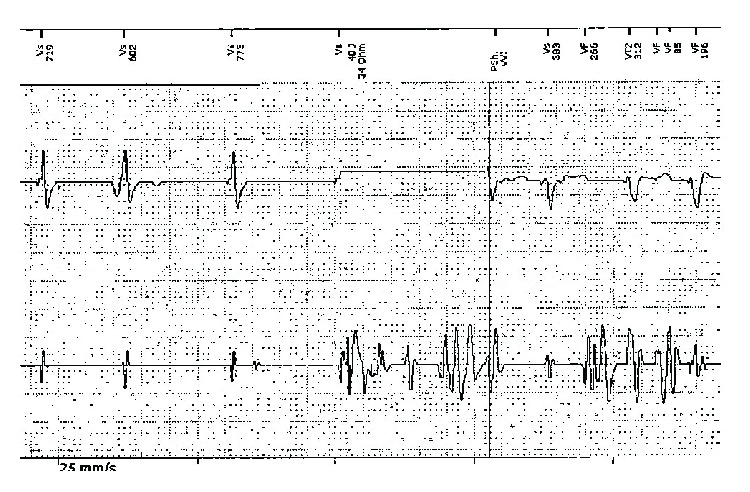
Delivery of 40-joule shock despite resolution of inappropriate VF sensing prior to the committed nature of therapy. Note that due to shock delivery, vigorous ventricular contraction occurs that resumes the lead chattering inappropriate sensing as VF (from top to bottom: marker channel, RV shock electrogram, and RV sense electrogram).

In earlier 2015, the patient presented with receipt of six ICD shocks without prior symptoms. Interrogation of the ICD disclosed intermittent, noncyclical, and nonphysiologic noise signals. The intracardiac electrogram recording was shown (Figure 1). All the device parameters were found within normal limits.

**Figure 2:**
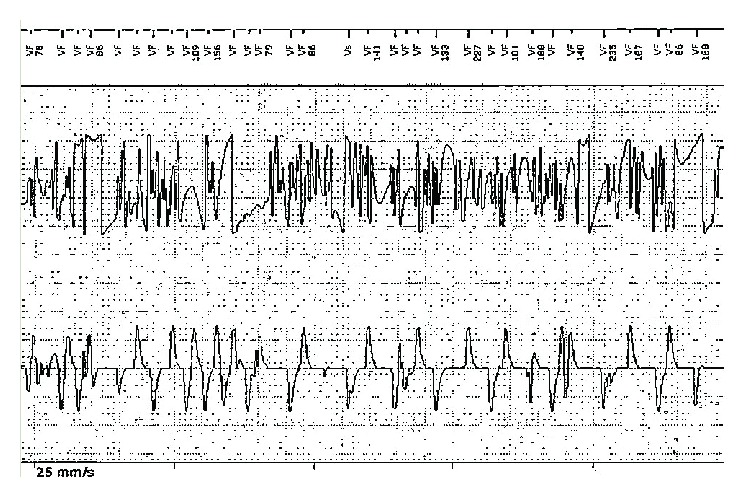
Intermittent nonphysiologic noise signals upon ICD interrogation (from top to bottom: marker channel, RV shock electrogram, and RV sense electrogram).

Lead failure was unlikely as all sensing and pacing parameters including lead impendence of the new Medtronic Sprint Quattro lead were normal. The signals (Figure 2) were noncyclical and very chaotic, ruling out the T-wave oversensing or crosstalk from far-field P-wave sensing. The noise signals were not suggestive of electromagnetic interference since external interferences are often repetitive high-frequency signals. The signals were not reproducible with isometric arm exercises. The patient was not physically active prior to or during the delivery of inappropriate shocks excluding the possibility of interference from pectoral myopotentials.

**Figure 3:**
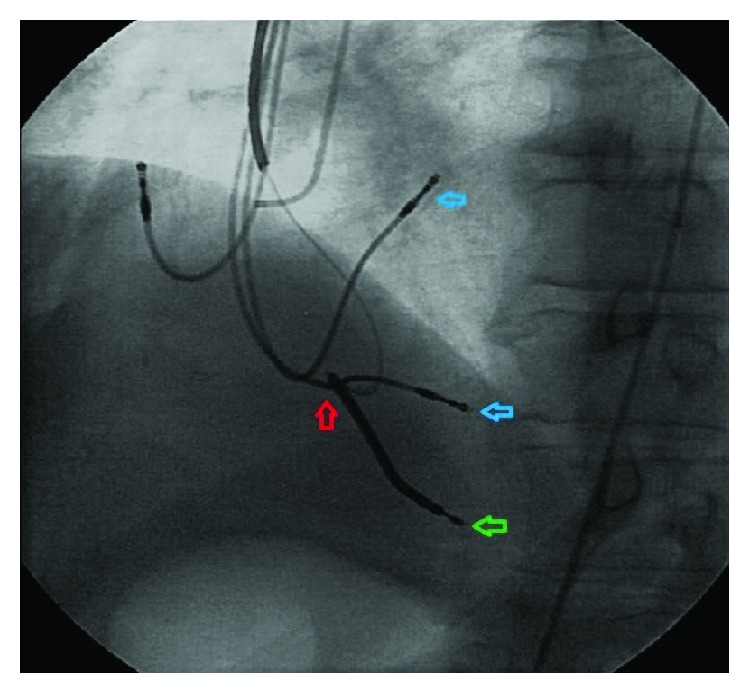
CCM signal-delivering leads in the upper and middle septum (blue arrows) and single chamber ICD lead at RV apex (green arrow) chattering each other at tricuspid annulus (red arrow).

The patient underwent fluoroscopic examination (Figure 3) on which right ventricular leads of the CCM unit were found to be chattering with the newly inserted dual-coil ICD lead at the tricuspid annulus. Intracardiac mechanical interference/lead chattering was the cause of inappropriate sensing to result in an inappropriate ICD firing. The intermittent noise signals seen on the device intracardiac electrogram were coincidental with the leads chattering on the fluoroscopy.

The CCM device along with two St. Jude Medical right ventricular pace-sense leads was extracted, but the same ICD unit was left in place. During the follow-ups over two years, no further noise signals had been detected by his ICD and the patient had not received any more inappropriate shocks.

## 3. Discussion

Cardiac contractility modulation (CCM) device is designed to enhance ventricular contractility in a subgroup of systolic heart failure patients who are not candidates for cardiac resynchronization therapy (CRT) owing to the absence of wide QRS complexes or who have failed to respond on CRT. Although CCM therapy falls under the investigational category in the United States, it is approved as one of the device-based therapy for heart failure in Europe. CCM therapy can provide a better quality of life and NYHA functional class by improving peak venous oxygen (pVO_2_), Minnesota Living with Heart Failure Questionnaires (MLWHFQ) in narrow QRS systolic heart failure patients despite its lack in mortality benefits or all-cause hospitalizations [1–3]. Also, improved myocardial contractility induced by CCM was not found in association with an increase in myocardial oxygen consumption [4, 5].

The Optimizer CCM system includes a pulse generator and three leads. Current available CCM devices use three pace-sense leads with one lead in the right atrium and two in the right ventricular septum with a minimum of 1-2 cm apart. The right atrial lead detects atrial signals and transmits to the pulse generator. Based on atrial signals and predetermined measurements, the pulse generator generates the CCM signals to the right ventricle during the absolute refractory period [6–8].

As the primary use of CCM is for systolic heart failure, most of the patients on CCM therapy have a concomitant ICD device. The manufacturer of Optimizer CCM declares that the system is designed to work in parallel with any CEIDs (cardiac electronic implantable devices) including ICDs and generally does not cause any interruption with ICD functions. However, functional testing of both Optimizer CCM and ICD systems is crucial and mandated to conduct during every procedure including the time of CCM implantation, an addition or revision of a lead(s) to ensure there is neither oversensing of CCM signals by the ICD nor any device-device interactions. It is important to allow an optimal spacing between the ICD lead and the CCM signal-delivering leads to prevent chattering each other in the RV.

To our best knowledge, the consequences of CCM-ICD leads chattering as an outcome of inappropriate delivery of ICD shocks which even warranted the extraction of the CCM device have never been reported. In our case, CCM and the old ICD units were working in parallel without any interactions until the ICD lead was replaced. The diagnosis was challenging since signals were similar to those of ICD lead dysfunction but the leads parameters were normal. Under fluoroscopic examination, it was found that all the leads were implanted 1-2 cm apart as recommended by the manufacturer. However, there was the excessive redundancy of some of the leads, and it was believed to be the etiology of leads chattering and oversensing by the ICD. Our case gives us a valuable lesson regarding the crucial role of CCM-ICD leads positioning and mandatory testing of device-device interactions during each and every CEID procedure.

## 4. Conclusion

Optimizer is an investigational device based on the concept of cardiac contractility modulation. It is designed to improve myocardial contractility without increasing cardiac oxygen consumption by facilitating the calcium handling of cardiomyocytes. The device has a potential to fill up the present therapeutic gap in the treatment of systolic heart failure patients who have narrow QRS complexes or failed to CRT. The majority of patients receiving CCM therapy have concurrent ICD and the manufacturer declared both devices can be used in parallel without any interaction in between. Nevertheless, proper leads positioning of both devices are crucial, and it is mandatory to check device-device interactions during each and every CEID procedure to prevent adverse outcomes.
